# An Accurate Non-Cooperative Method for Measuring Textureless Spherical Target Based on Calibrated Lasers

**DOI:** 10.3390/s16122097

**Published:** 2016-12-09

**Authors:** Fei Wang, Hang Dong, Yanan Chen, Nanning Zheng

**Affiliations:** Institute of Artificial Intelligence and Robotics, Xi’an Jiaotong University, Xi’an 710049, China; wfx@mail.xjtu.edu.cn (F.W.); chenyanan@stu.xjtu.edu.cn (Y.C.); nnzheng@mail.xjtu.edu.cn (N.Z.)

**Keywords:** non-cooperative target, pose estimation, sensor fusion, laser-camera system calibration, textureless target

## Abstract

Strong demands for accurate non-cooperative target measurement have been arising recently for the tasks of assembling and capturing. Spherical objects are one of the most common targets in these applications. However, the performance of the traditional vision-based reconstruction method was limited for practical use when handling poorly-textured targets. In this paper, we propose a novel multi-sensor fusion system for measuring and reconstructing textureless non-cooperative spherical targets. Our system consists of four simple lasers and a visual camera. This paper presents a complete framework of estimating the geometric parameters of textureless spherical targets: (1) an approach to calibrate the extrinsic parameters between a camera and simple lasers; and (2) a method to reconstruct the 3D position of the laser spots on the target surface and achieve the refined results via an optimized scheme. The experiment results show that our proposed calibration method can obtain a fine calibration result, which is comparable to the state-of-the-art LRF-based methods, and our calibrated system can estimate the geometric parameters with high accuracy in real time.

## 1. Introduction

Measurement for non-cooperative targets is the precondition of assembling and capturing tasks, which has received attention in various areas, such as autonomous robotics [1,2], marine transportation [3,4,5] and aerospace [6,7]. Non-cooperative targets refer to those objects that cannot provide effective cooperation information; their structure, size and motion information are completely or partly unknown [8].

In the measuring and capturing of a non-cooperative target, computer vision is exclusively used as the primary feedback sensor to acquire the pose information of the target. According to the number of cameras, vision measurement methods for non-cooperative targets can be classified into three types: monocular vision based, multi-vision based and multi-sensor fusion based. For the methods using monocular vision, Zhang et al. [8] proposed a robust algorithm based on Random Sample Consensus (RANSAC) to acquire the relative pose of a spacecraft. Fang et al. [9] presented a novel two-level scheme for adaptive active visual servoing to determine relative pose between a camera and a target. For the methods using multi-vision, Xu et al. [10] reconstructed the 3D model of non-cooperative spacecrafts and calculated the pose of the spacecraft based on stereo vision. In [11], Segal et al. employed a stereoscopic vision system for determining the relative pose of the non-cooperative spacecraft by tracking feature points on it. The camera-only methods always rely on the texture information of the target, which do not perform well with poorly-textured targets.

With the rapid development of multi-sensor fusion technology, camera-only based methods are gradually being replaced by multi-sensor fusion methods in the study of non-cooperative object measurement. To enhance the accuracy of pose estimation, a camera is combined with 2D or 3D laser scanners in [12,13], which can resolve the inaccuracy of depth in stereo-vision systems by directly measuring the depth of correspondence points. Myung et al. [14,15] proposed a structured light system that illuminates patterns of light to calculate the plane-to-plane relative position. This system is composed of two screen planes at both the system and target side, each having one or two laser pointers and a camera installed on the screen. The laser triangulation system (LTS) is another solution to accurate reconstruction for non-cooperative targets. Santolaria et al. [16] designed metrology equipment, which integrates a commercial LTS with an articulated arm coordinate measuring machine (AACMM) to extend the measurement range of LTS. However, it cannot be integrated with handheld devices and mobile robotic platforms due to the existence of AACMM.

Recently, many systems and applications that combine cameras and laser range finders (LRF) for non-cooperative target estimation and reconstruction have been widely applied in city model acquisition [17], object mapping [18,19,20], object tracking [21,22,23], augmented reality [24] and mobile robotics [18,19,20,21,22,23]. Atman et al. [25] developed a camera-LRF hybrid sensor, which can estimate the ego-motion of the micro air vehicles (MAVs) for the MAV’s navigation systems. Oh et al. [26] proposed a novel localization approach based on a hybrid method incorporating a 2D laser scanner and a monocular camera in the framework of a graph structure-based SLAM. Representatively, a non-contact six-DOF pose sensor system with three 1D laser sensors and a camera was developed to track the dynamic motions of a cargo ship [3,4,5]. This system can accurately measure a six-DOF pose from a distance by tracking feature points of the object.

The existing multi-sensor fusion methods need either expensive laser range finders [27] or delicate scanning laser triangulation systems [16]. They may be also limited with respect to portability because the screen-camera unit needs to be attached to the surface of the target [14,15]. Therefore, focusing on the application of industrial quality detection and object capturing tasks, we propose a much less expensive and fully-portable system for handheld devices and lightweight robotic platforms to measure the geometric parameters of textureless non-cooperative spheres at a near distance (<2 m). Furthermore, the aim of this study is to mitigate the limitations of the existing systems and to provide an inexpensive embedded solution for engineering applications.

Inspired by multi-sensor fusion methods, the proposed system is composed of four simple lasers and a vision camera, which can directly measure the position and radius of a textureless sphere accurately without any extra sensors on the target. Unlike camera-LRF and camera-LTS methods, we replace the widely-used laser range finder and laser triangulation system with four simple lasers to make our system more affordable and lightweight. A simple laser here means the simplest laser diode that can only project one point to the target without any direct depth information.

The reconstruction methods based on this camera-laser setup always require getting the relative pose between the camera and the laser in advance. Therefore, the calibration of such setups has attracted increasing attention from researchers. Whether requiring a pre-defined calibration object or not, these approaches can be roughly grouped into two categories: offline calibration and online calibration. The offline extrinsic calibration process of camera-LRF fusion systems has been discussed in published works [27,28,29,30,31]. The most well-known one is proposed by Zhang and Pless in [28]. They calibrated the extrinsic parameters with a planar calibration pattern, which can be viewed simultaneously by the camera-LRF system. Soon, Unnikrishnan and Hebert developed an easy-to-use software to calibrate the relative pose of 3D LRF [29]. The work in [31] proposed a self-calibration method used in the rotation platform. In [30], a minimal solution for extrinsic calibration is proposed by Vasconcelos and Barreto, which requires at least five planes. Most recently, Nguyen and Reitmayr [27] proposed two methods to calibrate a camera-LRF fusion system. As for the extrinsic calibration of the camera-LTS fusion system, Santolaria et al. [16] developed a one-step calibration method to obtain both the intrinsic—laser plane, CCD sensor and camera geometry—and extrinsic parameters of the LTS related to an articulated arm coordinate measuring machine (AACMM). Besides these offline extrinsic calibration methods, researchers also proposed many online extrinsic calibration methods [32,33,34,35] that can update the extrinsic parameters over time. The work in [32] provided an efficient and practical camera online calibration method that utilizes the lane markings for the tilt and pan angle calibration based on a zero roll angle assumption. The work in [33] exploited the line edges/features of handy objects to calibrate both the intrinsic and extrinsic parameters of the camera online, which provide a large degree of stability to illumination and viewpoint changes and offer some resilience to hash imaging conditions, such as noise and blur. However, benefiting from our all-in-one design in which the relative variation between the camera and simple lasers can be ignored, we choose the offline calibration method for better accuracy.

Obviously, all of the existing offline methods cannot be directly applied to calibrate the simple laser system. Thus, we propose an efficient method to calibrate the extrinsic parameters between a camera and a simple laser. While we share with [16] the same concept of the ray triangulation principle [36], our extrinsic calibration method differs in the following ways. In contrast to the one-step calibration methods whose intrinsic and extrinsic parameters between the camera and the laser come entirely from one calibration image, ours intrinsic parameters of the camera come from an optimized method that is more accurate. Moreover, our method is designed to determine the general equation of four laser beams instead of the laser plane, because of the simplicity of our laser system. Our experiments show that our method performs better than Nguyen and Reitmayr’s calibration result in [27] when using synthetic data.

By using the calibrated lasers and the camera projection model, our method can achieve a highly accurate result with three steps: (1) utilize the optical line constraint to reconstruct the 3D positions of laser spots; (2) obtain the initial guess of the geometric parameters via sphere fitting; (3) add a geometric constraint term to the final cost function, and optimize it to refine the initial guess. We conclude by giving both simulations and experimental results showing the success of the techniques presented. Comparing with existing frameworks, our scheme shows several advantages, including no requirement of the target texture information, no use of any depth sensor and no aid of other complicated equipment, such as LRF, LTS or articulated arm systems. Another feature of the proposed system is portability. All of the units are integrated as one sensor on the end-effector, without installing any sensor unit on the target. The performance of the proposed system has been validated in an embedded system with field experiments.

This paper is organized as follows: Section 2 describes our proposed calibration method. Section 3 shows how to reconstruct the laser spots with a calibrated laser-camera system and obtain the refined geometric parameters with an initial guess. We evaluate the calibration results and reconstruction solutions with simulation and field experiment in Section 4. Finally, some conclusions are drawn in Section 5.

## 2. Extrinsic Calibration

### 2.1. System Description

**Figure 1:**
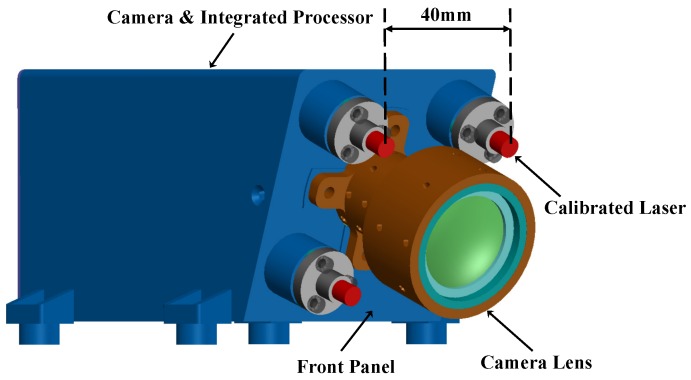
3D model of the measurement system.

As shown in Figure 1, the designed measurement system is composed of two parts: four calibrated lasers and a vision camera. Four lasers are placed on the front panel of the camera in a square configuration with a width of about 40 mm. The reason why we have chosen the configuration with four lasers will be discussed in the experiment section. The lens of the vision camera is installed in the center of the four lasers. The camera-laser system has the fundamental image preprocessing function, laser-detection algorithm and the measurement module embedded, which are necessary for calculating the geometric parameters of the target. Notice that our calibration method assumes that the accurate intrinsic matrix of the camera is obtained by Zhang’s algorithm [37], and the geometric parameters of each laser beam with respect to (w.r.t.) the camera coordinate frame are the unknown extrinsic parameters. The details of the extrinsic calibration will be discussed in Section 2.2 and Section 2.3. All the important symbols used in the following sections are listed in Table 1.

### 2.2. Description of the Calibration Coordinate Frame

**Figure 2:**
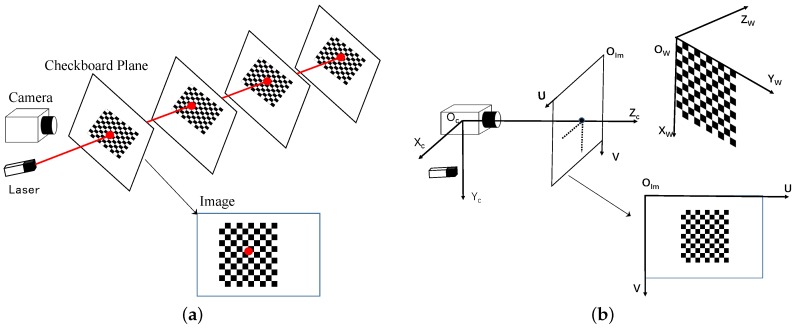
Illustration of the proposed calibration method: (**a**) design of the proposed calibration method; (**b**) diagram of the calibration reference coordinate frames.

Our goal in this section is to develop a way to determine the extrinsic parameters ${}^{c}L$ and ${}^{c}D$, which define the installation positions and direction vectors of all simple lasers w.r.t. camera coordinate frame {C}. During the calibration process, we only need a checkerboard plane, which will be moved several times to get an accurate calibration result. As shown in Figure 2a, several checkerboard settings are captured in our proposed method, and at each pose, the laser should fall on the checkerboard plane and form a spot.

The calibration system has three different coordinate frames: the world coordinate frame with its origin at the upper-left corner of the checkerboard; the camera coordinate frame with its origin at the optical center of the camera; the image coordinate frame with its origin at the top left corner of the image plane. A diagram of the coordinate frames is shown in Figure 2b.

### 2.3. Extrinsic Calibration Algorithm

This section shows our proposed calibration method for the extrinsic calibration of a camera and a simple laser. We assume that the intrinsic matrix of camera $M_{camera}$ is known and that the radial distortion has already been wrapped. The laser beam’s extrinsic parameters can be represented as the function of the laser beam with respect to the camera coordinate system:

(1) $$\left[\begin{array}{c} x\\ y\\ z \end{array}\right]={}^{c}D_{i}t_{i}+{}^{c}L_{i},$$

where $t_{i}$ is an arbitrary scale factor, ${}^{c}L_{i}={\left[{}^{c}x_{io},{}^{c}y_{io},{}^{c}z_{io}\right]}^{T}$ is the intersection point of laser beam *i* and image plane and ${}^{c}D_{i}={\left[m_{i},n_{i},p_{i}\right]}^{T}$ is the direction vector of laser beam *i* with respect to frame {C}.

We place the checkerboard at different poses. At each pose, the laser falls on the checkerboard plane and forms a spot. This laser spot’s coordinate is represented as ${}^{c}P_{i}={\left[{}^{c}p_{ix},{}^{c}p_{iy},{}^{c}p_{iz}\right]}^{T},i=\left\{a,b,c,d\right\}$ in the camera coordinate system. The function of the laser beam can be calculated if we get all of these laser spots’ coordinates. In order to get the coordinate of each laser spot, we utilize these two constraints at each different checkerboard pose:

- The laser spot is on the line that goes through the camera optical center and the laser spot. We call this the optical line for convenience.
- The laser spot is on the plane of the checkerboard.

Considering the first constraint, the optical line can be calculated as follows. We approximate the camera model by a pinhole model, then a projection from laser spot ${}^{c}P_{i}={\left[{}^{c}p_{ix},{}^{c}p_{iy},{}^{c}p_{iz}\right]}^{T}$ to 2D image coordinates ${}^{im}P_{i}={\left[{}^{im}p_{ix},{}^{im}p_{iy}\right]}^{T}$ can be given by:

(2) $${}^{im}P_{i}=\pi \left({}^{c}P_{i}\right)=M_{camera}{}^{c}P_{i},$$

where $M_{camera}$ is the intrinsic matrix of the camera and ${}^{im}P_{i}$ should be equal to the detected coordinate ${\tilde{p}}_{i}$ in the image. Then, the direction vector of the optical line that goes through ${}^{c}P_{i}$ can be represented as:

(3) $$D_{oi}=M_{camera}^{-1}\left[\begin{array}{c} {\tilde{p}}_{i}\\ 1 \end{array}\right].$$

Then, the optical line’s function can be given by:

(4) $$\left[\begin{array}{c} x\\ y\\ z \end{array}\right]=k_{i}D_{oi},$$

where $k_{i}$ is a scale factor and $D_{oi}$ is the direction vector of the optical line.

By substituting Equation (3) into Equation (4), we can derive:

(5) $${}^{c}P_{i}=k_{i}M_{camera}^{-1}\left[\begin{array}{c} {\tilde{p}}_{i}\\ 1 \end{array}\right].$$

Considering the second constraint, the checkerboard plane’s function can be calculated as follows. The transformation matrix $\left[{}_{c}^{w}R\;{}_{c}^{w}T\right]$, which relates the world coordinate system to the camera coordinate system, can be calculated by Zhang’s method [10]. Then, the normal vector of this plane can be represented as:

(6) $$N=-R_{3}\left(R_{3}^{T}T\right),$$

where $R_{3}$ is the third column of ${}_{c}^{w}R$. Therefore, the function of this plane is:

(7) $$\left[\begin{array}{c} N^{T}\;{∥N∥}_{2} \end{array}\right]\left[\begin{array}{c} {}^{c}P_{i}\\ 1 \end{array}\right]=0.$$

By substituting Equation (6) into Equation (7), the checkerboard plane’s function can be represented as:

(8) $$\left[\begin{array}{c} -R_{3}^{T}\left(R_{3}T^{T}\right)\;{∥-R_{3}\left(R_{3}^{T}T\right)∥}_{2} \end{array}\right]\left[\begin{array}{c} {}^{c}P_{i}\\ 1 \end{array}\right]=0.$$

Utilizing the two constraints mentioned above, we can get the coordinate of ${}^{c}P_{i}$ by combining Equations (5) and (8). Since we move the checkerboard plane several times, a series of 3D coordinates of laser spots $\tilde{{}^{c}P_{i}}=\left\{{}^{c}P_{i1},{}^{c}P_{i2},\cdots ,{}^{c}P_{in}\right\}$ can be acquired. Assuming that the lasers are fixed, these spots should be on the same line. Then, the function of the laser beam can be determined using these points. In order to get the optimal parameters of the laser beam, we use PCA to minimize the projection error of all of these spots:

- First, calculate the center point of all of the laser spots $\bar{{}^{c}P_{i}}=\frac{sum(\tilde{{}^{c}P_{i}})}{n}$.
- Second, normalize all of the laser spots $\hat{{}^{c}P_{i}}=\frac{\tilde{{}^{c}P_{i}}-\bar{{}^{c}P_{i}}}{max(\tilde{{}^{c}P_{i}})}$.
- Third, compute the covariance matrix $\Sigma =\frac{{\hat{{}^{c}P_{i}}}^{T}\hat{{}^{c}P_{i}}}{n}$, and compute the eigenvectors of the covariance matrix [USV]=svd(Σ).
- Then, the direction vector of laser beam *i* can be $\bar{{}^{c}D_{i}}=U\left(:,1\right)$.

Therefore, laser beam *i*’s function is:

(9) $$\left[\begin{array}{c} x\\ y\\ z \end{array}\right]=\bar{{}^{c}D_{i}}t_{i}+\bar{{}^{c}P_{i}}.$$

However, the parameters of this function are not unique. In order to disambiguate, we transform this function to another equivalence form. The direction vector $\bar{{}^{c}D_{i}}$ will be replaced by ${}^{c}D_{i}=\frac{\bar{{}^{c}D_{i}}}{∥\bar{{}^{c}D_{i}}∥}$, and point $\bar{{}^{c}P_{i}}$ will be replaced by ${}^{c}L_{i}={\left[{}^{c}l_{ix}\;\;{}^{c}l_{iy}\;\;0\right]}^{T}$, which is the intersection point of the laser beam and the image plane. Thus, the final result is:

(10) $$\left[\begin{array}{c} x\\ y\\ z \end{array}\right]=\frac{\bar{{}^{c}D_{i}}}{∥\bar{{}^{c}D_{i}}∥}t_{i}+\left[\begin{array}{c} {}^{c}l_{ix}\\ {}^{c}l_{iy}\\ 0 \end{array}\right].$$

## 3. Measurement Algorithm Description

**Figure 3:**
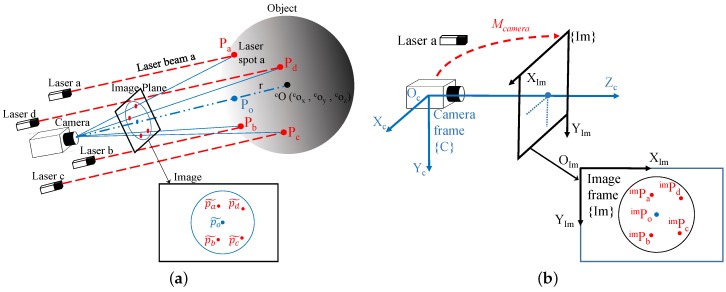
Illustration of the measurement method: (**a**) design of the measurement method; (**b**) diagram of the measurement reference coordinate frames.

Once all of the extrinsic parameters of simple lasers are calibrated, our system can achieve a highly accurate measurement of the spherical target with three steps: (1) reconstruct the 3D positions of laser spots; (2) obtain the initial guess of the solution via sphere fitting; (3) refine the initial guess by nonlinear optimization. An illustration of the proposed measurement method is shown in Figure 3a.

### 3.1. Description of the Reconstruction Coordinate Frame

The measurement system has two different coordinate frames: {C} is the camera coordinate frame with its origin at the center of the camera aperture. {Im} is the image coordinate frame with its origin at the top left corner of the image plane. The relationship between the camera coordinate frame and the image coordinate frame can be described by a pinhole model. All of these coordinate frames are orthogonal. The principle of measuring an unknown spherical target is solving for the geometric parameters: ${}^{c}O={\left[{}^{c}o_{x},{}^{c}o_{y},{}^{c}o_{z}\right]}^{T}$, the 3D position of the sphere center with respect to frame {C}, and *r*, the radius of the sphere. A diagram of the coordinate frames is shown in Figure 3b.

### 3.2. Initial Guess of Geometric Parameters

In order to calculate the parameters of an unknown sphere, at least four non-coplanar points on the surface of the sphere are needed. As shown in Figure 3a, the laser spot should satisfy the following two constraints:

- The laser spot is on the optical line.
- The laser spot is on the laser beam that has been calibrated in the prior section.

Considering the first constraint, we firstly detect the laser spot *i*’s pixel coordinate ${\tilde{p}}_{i}={\left[u\;\;v\right]}^{T}$ in the image. Then, the function of the optical line can be calculated by the approach described in the last section. We represent this line as:

(11) $$\left[\begin{array}{c} x\\ y\\ z \end{array}\right]=k_{i1}D_{oi},$$

where $D_{oi}$ is determined by Equation (3).

Considering the second constraint, the function of the laser beam *i* can be represented as:

(12) $$\left[\begin{array}{c} x\\ y\\ z \end{array}\right]=k_{i2}{}^{c}D_{i}+{}^{c}L_{i},$$

where ${}^{c}D_{i}$ and ${}^{c}L_{i}$ can be determined by our proposed calibration method.

Then, we can reconstruct laser spot *i*’s coordinate by utilizing these two constraints: laser spot *i* should be the intersection of these two lines. Combining Equations (11) and (12), laser spot *i*’s 3D position can be recovered using the least square method. It is equivalent to minimizing:

(13) $${∥k_{i1}D_{oi}-\left(k_{i2}{}^{c}D_{i}+{}^{c}L_{i}\right)∥}_{2},$$

where $k_{1},k_{2}$ can be given by:

(14) $$\left[\begin{array}{c} k_{i1}\\ k_{i2} \end{array}\right]=-{\left(\left[\begin{array}{c} D_{oi}^{T}\\ {}^{c}D_{i}^{T} \end{array}\right]\left[\begin{array}{c} D_{oi}\;\;{}^{c}D_{i} \end{array}\right]\right)}^{-1}\left[\begin{array}{c} D_{oi}^{T}\\ {}^{c}D_{i}^{T} \end{array}\right]{}^{c}L_{i}.$$

Therefore, the reconstruction result of laser spot i can be given by:

(15) $${}^{c}P_{i}=\frac{1}{2}\left(k_{i1}D_{oi}+k_{i2}{}^{c}D_{i}+{}^{c}L_{i}\right).$$

With four constructed laser spots, the geometric parameters $\left[{}^{c}O_{0},r_{0}\right]$ of the target can be determined by sphere fitting. However, because every four non-coplanar points will determine a sphere, the accuracy of sphere fitting will be greatly affected by the reconstruction error of laser spots. Therefore, we should use the solution from four reconstruction points as the initial guess and refine it with nonlinear optimization by adding the projection point of the center of the sphere as a geometric constraints.

### 3.3. Nonlinear Optimization

To achieve a more accurate solution, we will utilize an optimized scheme for each frame by minimizing the combination of reprojection errors of laser spots and the center of the sphere as follows:

(16) $$\underset{{}^{c}O,\;r}{minimize}\sum_{i}{∥\pi \left(\Phi_{i}\left({}^{c}O,r,D_{i},L_{i}\right)\right)-{\tilde{p}}_{i}∥}_{2}+\lambda {∥\pi \left({}^{c}O\right)-{\tilde{p}}_{o}∥}_{2},$$

where *λ* is a tuning parameter and ${\tilde{p}}_{i}$, ${\tilde{p}}_{o}$ are the image coordinates of the detected laser spot *i* and the center of the projected circle as shown in Figure 3a.

The first term in the cost function Equation (16) is meant for penalizing the reprojection error of four laser spots, in which the function π() is the projection function and $\Phi_{i}\left({}^{c}O,r,D_{i},L_{i}\right)$ is the reconstruction function for each laser spot. As mentioned before, the reconstruction error of laser spots will lead to an inaccurate solution. To improve the robustness of the measurement system, we add a geometric prior term, which enforces the projection point of the optimized ${}^{c}O$ coinciding with the detected center of projected circle ${\tilde{p}}_{o}$. We minimize Equation (16) as a nonlinear optimization problem by using the Levenberg–Marquardt method [18,20,21]. This requires an initial guess of ${}^{c}O_{0}$ and $r_{0}$, which is obtained by using the method described in Section 3.2. In the following part of this section, the derivation of $\Phi_{i}\left({}^{c}O,r,D_{i},L_{i}\right)$ and π() will be given in detail.

#### 3.3.1. Formulation of the Reconstruction Function

Unlike the deduction process in Section 3.2, the $\Phi_{i}\left({}^{c}O,r,D_{i},L_{i}\right)$ is determined by another two constraints:

- The laser spot is on the surface of the target sphere.
- The laser spot is on the laser beam that has been calibrated in the prior section.

Assuming ${}^{c}P_{i}={\left[{}^{c}p_{ix},{}^{c}p_{iy},{}^{c}p_{iz}\right]}^{T},i=\left\{a,b,c,d\right\}$ is the 3D position of laser spot *i* on the target surface, its coordinate should satisfy the following formula of the sphere:

(17) $${\left({}^{c}p_{ix}-{}^{c}o_{x}\right)}^{2}+{\left({}^{c}p_{iy}-{}^{c}o_{y}\right)}^{2}+{\left({}^{c}p_{iz}-{}^{c}o_{z}\right)}^{2}=r^{2}.$$

Meanwhile, laser spot *i* is also restricted by the linear equation of laser beam *i*. The linear constraint can be given as follows:

(18) $$\left[\begin{array}{c} {}^{c}p_{ix}\\ {}^{c}p_{iy}\\ {}^{c}p_{iz} \end{array}\right]={}^{c}D_{i}t_{i}+{}^{c}L_{i},$$

where $t_{i}$ is an arbitrary scale factor. In this equation, ${}^{c}L_{i}={\left[{}^{c}x_{io},{}^{c}y_{io},{}^{c}z_{io}\right]}^{T}$ and ${}^{c}D_{i}={\left[m_{i},n_{i},p_{i}\right]}^{T}$ are calibrated by using the proposed method in Section 2.

Combining Equations (17) and (18), a quadratic equation of $t_{i}$ can be given as follows:

(19) $$Q_{sphere}\left[\begin{array}{c} t_{i}^{2}\\ 2t_{i}\\ 1 \end{array}\right]=0,$$

where:

$$\begin{array}{c} Q_{sphere}=\left[\begin{array}{c} q_{11},q_{12},q_{13} \end{array}\right]=\\ {\left[\begin{array}{c} m_{i}^{2}+n_{i}^{2}+p_{i}^{2}\\ m_{i}\left({}^{c}x_{io}-{}^{c}o_{x}\right)-n_{i}\left({}^{c}y_{io}-{}^{c}o_{y}\right)+p_{i}\left({}^{c}z_{io}-{}^{c}o_{x}\right)\\ {\left({}^{c}x_{io}-{}^{c}o_{x}\right)}^{2}+{\left({}^{c}y_{io}-{}^{c}o_{y}\right)}^{2}+{\left({}^{c}z_{io}-{}^{c}o_{x}\right)}^{2}-r^{2} \end{array}\right]}^{T}. \end{array}$$

Considering that the laser spot cannot be located on the the far side of the sphere, the only reasonable solution of $t_{i}$ can be easily solved from Equation (19):

(20) $$t_{i}=\frac{-q_{12}-\sqrt{q_{12}^{2}-q_{11}q_{13}}}{q_{12}}.$$

Finally, by substituting Equation (20) into Equation (18), the reconstructed 3D coordinate of laser spot *i* with respect to frame {C} can be represented as follows:

(21) $${}^{c}P_{i}=\Phi_{i}\left({}^{c}O,r,D_{i},L_{i}\right)=\left[\begin{array}{c} \begin{array}{c} {}^{c}x_{io}-\frac{m_{i}\left(q_{12}+\sqrt{q_{12}^{2}-q_{11}q_{13}}\right)}{q_{12}}\\ {}^{c}y_{io}+\frac{n_{i}\left(q_{12}+\sqrt{q_{12}^{2}-q_{11}q_{13}}\right)}{q_{12}}\\ {}^{c}z_{io}-\frac{p_{i}\left(q_{12}+\sqrt{q_{12}^{2}-q_{11}q_{13}}\right)}{q_{12}} \end{array} \end{array}\right].$$

Since the installation position ${}^{c}L_{i}$ and the direction vector ${}^{c}D_{i}$ of the laser sensor are determined by Equation (10), the 3D position of each laser spot only depends on the geometric parameters of the sphere ${\left[{}^{c}o_{x},{}^{c}o_{y},{}^{c}o_{z},r\right]}^{T}$.

#### 3.3.2. Formulation of the Reprojection Point

In order to solve the geometric parameters of sphere, the perspective projection relationship is used to describe the relationship between the 3D position of laser spot *i* and its pixel coordinate.

With camera projection matrix $M_{camera}$, the 3D position of laser spot *i* with respect to frame {C} can be warped into the pixel coordinate of frame {Im}, ${}^{im}P_{i}={\left[{}^{im}p_{ix},{}^{im}p_{iy}\right]}^{T}$. The reprojection coordinate of laser spot *i* can be expressed as follows:

(22) $$\pi \left(\Phi_{i}\left({}^{c}O,r,D_{i},L_{i}\right)\right)=\left[\begin{array}{c} {}^{im}p_{ix}\\ {}^{im}p_{iy}\\ 1 \end{array}\right]=M_{camera}\left[\begin{array}{c} \left.{}^{c}p_{ix}/{}^{c}p_{iz}\right.\\ \left.{}^{c}p_{iy}/{}^{c}p_{iz}\right.\\ \left.{}^{c}p_{iz}/{}^{c}p_{iz}\right.\\ \left.1/{}^{c}p_{iz}\right. \end{array}\right],$$

where:

(23) $$M_{camera}=\left[\begin{array}{cccc} f_{x} & 0 & c_{x} & 0\\ 0 & f_{y} & c_{y} & 0\\ 0 & 0 & 1 & 0 \end{array}\right],$$

and ${}^{c}p_{iz}$ is the depth of laser spot *i* in the frame {C}.

By substituting ${}^{c}p_{iz}$ in Equation (21) into Equation (22), the complete formulation of $\pi (\Phi_{i}\left({}^{c}O,r,D_{i},L_{i}\right))$ in the first term is determined. Obviously, the projected point ${}^{im}P_{i}={\left[{}^{im}p_{ix},{}^{im}p_{iy}\right]}^{T}$ should coincide with detected coordinate ${\tilde{p}}_{i}$ in the image, thus formulating the first term in Equation (16). According to the derived ${}^{c}P_{i}$ in Equation (21), the only unknown values in this function are the geometric parameters ${}^{c}O$ and *r*, which can be optimized with no less than four detected spots.

Furthermore, in order to restrain the effect of the reconstruction error, the reprojection coordinate of the center of the sphere is also applied:

(24) $$\pi \left({}^{c}O\right)=\left[\begin{array}{c} {}^{im}p_{ox}\\ {}^{im}p_{oy}\\ 1 \end{array}\right]=M_{camera}\left[\begin{array}{c} \left.{}^{c}o_{x}/{}^{c}o_{z}\right.\\ \left.{}^{c}o_{y}/{}^{c}o_{z}\right.\\ \left.{}^{c}o_{z}/{}^{c}o_{z}\right.\\ \left.1/{}^{c}o_{z}\right. \end{array}\right].$$

In the pinhole model, the reprojection point ${}^{im}P_{o}={\left[{}^{im}p_{ox},{}^{im}p_{oy}\right]}^{T}$ should coincide with the center of its projected circle ${\tilde{p}}_{o}$ in the image. Thus, the geometric term in Equation (16) is built.

By substituting Equations (22) and (24) into Equation (16), a more precise and robust solution of geometric parameters can be calculated by optimization.

It is obvious that our method can be easily extended to measure targets of different shapes, such as planes [3,4,5], spheroids and paraboloids, just by replacing the geometric function of the target Equation (17).

### 3.4. Algorithm Summary

The complete algorithm in this paper can be concluded as the following steps:

1. Use the checkerboard, and place it in front of the camera-laser system in different orientations to calibrate the intrinsic and extrinsic parameters of the system.
2. Take an image with the target, and detect the laser spots and the center of the projected circle.
3. Estimate the geometric parameters ${}^{c}O_{0}$ and $r_{0}$ using the method described in Section 3.2.
4. Build the cost function Equation (16) with the derivation in Section 3.3, and optimize ${}^{c}O$ and *r* by using the Levenberg–Marquardt method.

## 4. Experimental Results

According to the proposed framework, the experiment will be divided into three parts: the simulation of extrinsic calibration, the simulation of target measurement and the field experiment. First, we evaluate the robustness of our calibration algorithm by adding detection noise and more calibration poses. Then, we evaluate the accuracy of our geometric measurement method by taking the calibration errors of the laser beam into consideration. Finally, field experiments are conducted to evaluate the performance of the proposed system with the embedded platform.

### 4.1. Simulation of Extrinsic Calibration

In this section, we design a series of simulation experiments to validate the performance of our proposed calibration method. In order to represent a realistic measuring environment, the extrinsic parameters of the simulating laser *i* are defined as:

(25) $$\left\{\begin{array}{cc} {}^{c}D_{i} & =[-5,-5,100]\\ {}^{c}L_{i} & =[40\mathrm{mm},40\mathrm{mm},0]\\ {}_{c}^{w}T_{z} & \in [200\mathrm{mm},1200\mathrm{mm}] \end{array}\right.,$$

where ${}_{c}^{w}T_{z}$ is the depth of the checkerboard in the frame {C}. The camera’s intrinsic matrix is generated according to a real camera with resolution 1024×1024, and the radial distortion is set to zero.

The ground truth is generated with the following rules. The checkerboard plane is defined as 12×12 square grids, and the length of every square is 20 mm. It is placed at a limited distance from 200 mm to 1200 mm. At each distance, we randomize the angle of the checkerboard in the range of $\left[-20^{\circ },20^{\circ }\right]$ and the translation in the range of [−20mm,20mm]. Then, we calculate the intersection point of the laser beam and the checkerboard plane at each place. Finally, we calculate the reprojection point of the checkerboard grid and the laser spot according to the generated angle and translation.

To check the robustness of our proposed calibration method, Gaussian noise, with a mean of zero and a standard deviation of one pixel, is added to each reprojection of the checkerboard corner and laser spot. For different magnitudes of noises validation, we scale the default standard deviations by a factor in the range of [0.25,3.0].

The calibration result is calculated by the method we proposed. We compare the result with the ground truth. The direction error is measured by the absolute angle error between our result and the ground truth in degrees. The intersection point error is measured by the Euclidean distance between our result and the ground truth. We evaluate the proposed method in two different conditions:

- Different magnitudes reprojection noises with the same amount of poses.
- Different numbers of poses with the same magnitude of reprojection noise.

**Figure 4:**
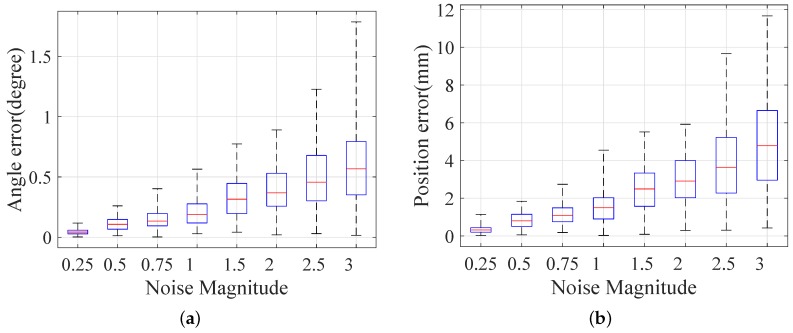
Error distribution under noise levels in the range of [0.25, 3.0]: (**a**) angular error of direction; (**b**) position error.

**Figure 5:**
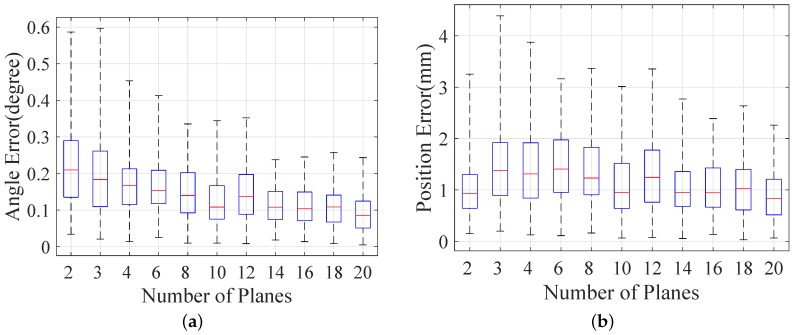
Error distribution under different numbers of poses in the range of [2, 20]: (**a**) angular error of direction; (**b**) position error.

We run 100 trials for every different magnitude noise and every different number of poses. First, we evaluate the effect of different reprojection noise with three poses. The standard deviation of Gaussian noise is one pixel, and it is scaled by a factor in the range of [0.25,3.0] in our simulations. The result is shown in Figure 4. Then, our method is evaluated under the second condition, and the number of poses is in the range of [2, 20]. The result is shown in Figure 5.

Figure 4 shows that the errors grow respectively with the noise magnitude, as expected. Compared to Nguyen and Reitmayr’s result in [27], our proposed method outperforms the baseline method by a more accurate result in terms of direction and position. Figure 5 shows that the error decreases along with the increasing number of planes. Nguyen and Reitmayr’s method reaches an acceptable level (below $10^{-2}$ m in position and around $10^{-1}$ in angle) with more than 10 planes. Our method provides a much better result in position (below 3 mm) and a comparable result in direction.

### 4.2. Simulation of Target Measurement

In this section, we design a series of simulations to validate the performance of our measurement system. In order to represent a realistic measuring environment, the measurement scenario is designed as follows:

(26) $$\left\{\begin{array}{cc} r & \in [30\;\mathrm{mm},100\;\mathrm{mm}]\\ {}^{w}O_{z} & \in [200\;\mathrm{mm},1200\;\mathrm{mm}]\\ {}^{w}O_{x},{}^{w}O_{y} & \in [-20\;\mathrm{mm},-20\;\mathrm{mm}] \end{array}\right..$$

Four lasers are installed in a square configuration with a width of about 60 mm, and four laser beams converge to the center of the square with an angle of $1^{\circ }$. In order to simulate a realistic measuring environment, random noises are added to the extrinsic parameters in the simulation: random variation of the $\left[-0.1^{\circ },0.1^{\circ }\right]$ angle error to converge to an angle and [−1 mm, 1 mm] position error to ${}^{c}L_{i}$. According to the repeated trials, the *λ* in the cost function Equation (16) is set to 60, which gives the minimum average error of all of the trials.

**Figure 6:**
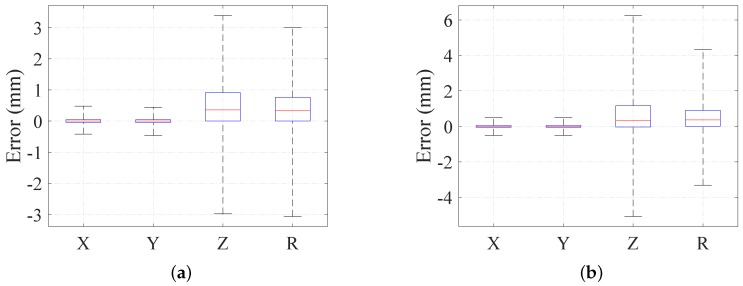
Simulation of the pose errors over a 500-mm distance with random noise levels of: (**a**) 0.5 pixels; and (**b**) one pixel.

In reality, the detection of laser spots can be influenced by the inappropriate exposure parameter and image noise, which will introduce random noises in the calculation. To ascertain the effects of noise on the proposed system, two different levels of random noises are added in ${}^{im}P_{i}$, respectively: random variation of the [−0.5,0.5] pixel error and the [−1,1] pixel error. After taking noises into account, the geometric parameters are calculated for the simulated spheres with a radius of around 60 mm. We randomly place the simulated sphere at 2000 different positions over a distance of 500 mm. The results for all of the noise levels are shown with the boxplot. As shown in Figure 6, the maximum absolute errors of position and radius in the noise simulation are less than 3.4 mm and 3 mm, for an added noise of 0.5 pixels. The errors increase to 6.3 mm and 4.3 mm at higher pixel noises.

**Figure 7:**
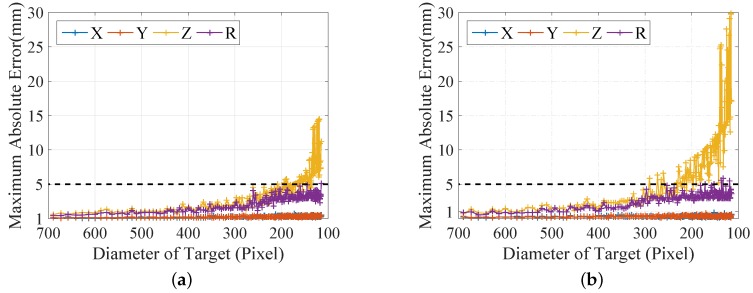
Simulation of the pose errors with different pixels of the diameter: (**a**) result with 0.5 pixels of noise; (**b**) result with one pixel of noise.

It is known that the accuracy of pose estimation for the cooperative target has a strong relationship with the distance. However, our target is non-cooperative, which means the accuracy is influenced by the distance and the size of the target simultaneously. Thus, the pixel of the diameter of the target is used to represent the effective measuring range of our system. We repeat the simulation 2000 times, which randomizes the radius and positions of the simulated sphere within the designed scenario and calculates its geometric parameters with noises added. The statistics of the maximum absolute errors under different pixels of the diameter are shown in Figure 7.

The results in Figure 7 show that: (1) the performance of our system slightly decreases as the pixel of the diameter decreases at first and drops dramatically when the diameter is lower than 300 pixels; (2) the maximum absolute errors of our system are less than 5 mm if the diameter is larger than 200 pixels for an added noise of 0.5 pixels; the lower bound of the diameter increases to 300 pixels for a higher noise of one pixel; (3) a better performance may be achieved by implementing the more robust laser spot detection method and a high quality camera with a higher resolution.

**Figure 8:**
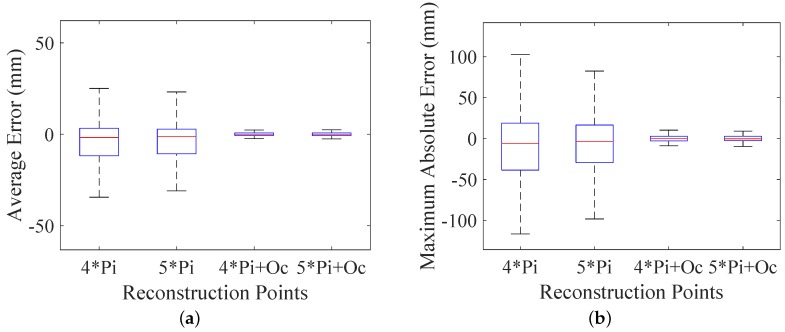
Pose error using different numbers of reconstruction points: (**a**) angular error of direction; (**b**) position error.

Finally, in order to validate the improvement of applying geometric prior term, optimizations without the geometric prior term are simulated to compare with the proposed method. In this comparison simulation, we successively use four laser spots, five laser spots, four laser spots with the geometric prior term and five laser spots with the geometric prior term to optimize the geometric parameters at 1000 different positions. The statistics of the average errors and maximum absolute errors are shown in Figure 8.

The results show that the optimizations with geometric prior term perform much better than the other two groups in both average errors and maximum absolute errors. The gradual improvement from the first boxplot to the third boxplot proves that the more reconstruction points are used in optimization, the more accuracy can be expected, while introducing the constraint of the coincidence between $\pi ({}^{c}O_{o})$ and ${\tilde{p}}_{o}$, which can significantly improve the performance (more than a ten-fold improvement) of the measurement system. This improvement validates the advantage of applying the geometric prior term. Moreover, the minor improvement between the third boxplot and the fourth boxplot shows that the number of reconstruction points is no longer the dominant factor of accuracy improvement when the geometric prior is already considered. That is the reason why the four-laser configuration is chosen as our final design (Figure 1), which retains the simplicity of the design while offering one-laser redundancy to ensure the robustness of the system.

### 4.3. Field Experiment

**Figure 9:**
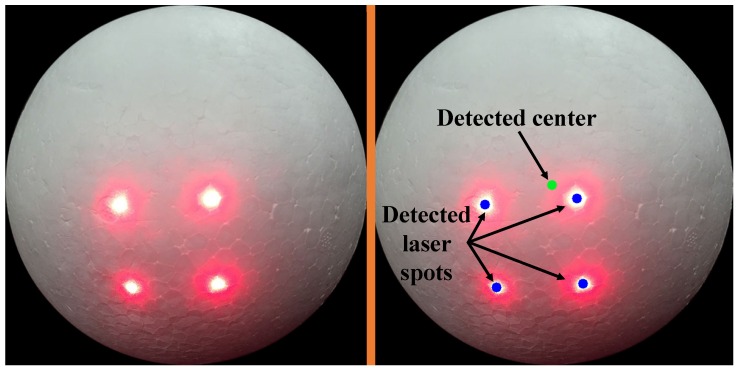
The image of the target sphere (**left**) and detected laser spots and the center point (**right**).

The performance of the proposed system is evaluated by conducting field experiments in which targets are placed at different positions from 200mm to 2000 mm in indoor environments. The tested system is implemented with an industry camera and four low-powered simple lasers and is fixed to a flat platform. The four lasers are set in a square configuration with a width of about 40 mm and a $1^{\circ }$ converge angle. The targets are a series of textureless white spheres with different radii (50 mm to 200 mm), as shown in Figure 9.

Before the test, the intrinsic and extrinsic parameters of the laser-camera system are calibrated by using Zhang’s algorithm [37] and the method proposed in Section 2 with 10 checkerboard poses. The image processing, spot detection and other numerical calculations can be done in real time with a XC4VSX55 FPGA and a TMS320C6701 DSP integrated in the camera. The acquired images are used to detect the laser spots and the center of the circle for the geometric parameters’ optimization in Equation (16). To obtain the ground truth of the target, we establish a precision measurement system with two Leica TM6100A theodolites. First, a calibration board is used as an intermediate coordinate to acquire the relative position between the theodolite coordinate frame and frame {C}. Then, at each trial, we acquire the position of six points on the target surface w.r.t. the theodolite coordinate frame via two theodolites. Finally, the ground truth of the geometric parameters w.r.t. {C} is calculated with data processing software.

After running 200 trials, the overall performance is evaluated. The maximum absolute errors of position and radius are 4 mm and 3.8 mm, respectively, which validate the accuracies of our proposed calibration method and the measurement framework. Furthermore, our measurement system also shows good performance in estimating the position of the spherical-like target, such as a polyhedron: The overall accuracy for the polyhedron with 26 facets in the same field experiment is 8 mm, which shows the generality and flexibility of our system. The experiment results show that the performance of the proposed system is comparable to other state-of-the-art multi-sensor methods. A detailed comparison of multi-DOF sensors for measurement applications is summarized in Table 2.

## 5. Conclusions

In this paper, a novel vision measurement system with four simple lasers is proposed to accurately calculate the geometric parameters of textureless non-cooperative spherical targets. With the efficient extrinsic calibration method of the laser-camera system proposed in this paper, our system can achieve an accurate solution of geometric parameters via an optimized scheme in real time. Compared to other systems, the proposed system requires neither the geometry information nor the texture information of the target in advance and is suitable for a variety of engineering occasions because of its simplicity, portability and low-power consumption.

Our simulation shows that our calibration method can provide an accurate result, which is comparable to the state-of-the-art LRF-based methods and can ensure 3.4-mm accuracy when recovering the geometric parameters of a spherical target with 0.5 pixels of detection noise added. The simulation results also prove that the proposed geometric prior term largely improves the accuracy of reconstruction.

Field experiments conducted within the designed scenario demonstrate that the overall performance of the system corresponds to accuracies of 4 mm and 3.8 mm for the position and radius and still ensures 8-mm accuracy when the target switches to a polyhedron with 26 facets.

Another advantage of this method is that it can be easily extended to targets with different shapes, just by replacing the target geometric function Equation (17) and installing more lasers to meet the minimal requirement of reconstruction points if necessary.

In future work, a new algorithm should be developed to measure the geometric parameters of the target with an unknown curved surface and shape, and we are also intent to seek for more joint applications in the SLAM and AR fields.

## Figures and Tables

**Table 1:** List of symbols.

{W}	World coordinate frame
{C}	Camera coordinate frame
{Im}	Image coordinate frame
$M_{camera}$	Camera projection matrix
${}^{c}L_{i}$	Installation position of laser *i* w.r.t. {C}
${}^{c}D_{i}$	Direction vector of laser beam *i* w.r.t. {C}
$D_{o}$	Direction vector of the optical line
*r*	Radius of the sphere
${}^{c}P_{i}$	Position of laser spot *i* w.r.t. {C}
*π*	Projection function of the vision camera
${}^{im}P_{i}$	Reprojection coordinate of laser spot *i*
${}^{im}P_{o}$	Reprojection coordinate of the center of the projected circle
${\tilde{p}}_{i}$	Image coordinate of the detected laser spot *i*
${\tilde{p}}_{o}$	Image coordinate of the detected center of the projected circle

**Table 2:** Comparison of multi-DOF sensors for measurement applications.

Method	Accuracy	Remark
Proposed System	<4 mm	based on simple lasers and camera
Three-Beam Detector [3]	<3 mm	installation of a camera on the target
Portable Three-Beam Detector [5]	<4 mm	based on 1D LRFs and camera
Handheld Camera-Laser System [13]	∼20 mm	based on 2D laser scanners and Camera
Laser 2D Scanner [12]	∼60 mm	sub-cm accuracy
Single-point 1D Laser Sensor [38]	∼12 mm	based on single-point LRFs
Laser Tracker [39]	∼15 μm	high cost
